# Patterns of Media Use, Strength of Belief in COVID-19 Conspiracy Theories, and the Prevention of COVID-19 From March to July 2020 in the United States: Survey Study

**DOI:** 10.2196/25215

**Published:** 2021-04-27

**Authors:** Daniel Romer, Kathleen Hall Jamieson

**Affiliations:** 1 Annnenberg Public Policy Center University of Pennsylvania Philadelphia, PA United States

**Keywords:** COVID-19, conspiracy beliefs, social media, print news media, broadcast news media, conservative media, vaccination, mask wearing, belief, misinformation, infodemic, United States, intention, prevention

## Abstract

**Background:**

Holding conspiracy beliefs regarding the COVID-19 pandemic in the United States has been associated with reductions in both actions to prevent the spread of the infection (eg, mask wearing) and intentions to accept a vaccine when one becomes available. Patterns of media use have also been associated with acceptance of COVID-19 conspiracy beliefs. Here we ask whether the type of media on which a person relies increased, decreased, or had no additional effect on that person’s COVID-19 conspiracy beliefs over a 4-month period.

**Objective:**

We used panel data to explore whether use of conservative and social media in the United States, which were previously found to be positively related to holding conspiracy beliefs about the origins and prevention of COVID-19, were associated with a net increase in the strength of those beliefs from March to July of 2020. We also asked whether mainstream news sources, which were previously found to be negatively related to belief in pandemic-related conspiracies, were associated with a net decrease in the strength of such beliefs over the study period. Additionally, we asked whether subsequent changes in pandemic conspiracy beliefs related to the use of media were also related to subsequent mask wearing and vaccination intentions.

**Methods:**

A survey that we conducted with a national US probability sample in March of 2020 and again in July with the same 840 respondents assessed belief in pandemic-related conspiracies, use of various types of media information sources, actions taken to prevent the spread of the disease and intentions to vaccinate, and various demographic characteristics. Change across the two waves was analyzed using path analytic techniques.

**Results:**

We found that conservative media use predicted an increase in conspiracy beliefs (β=.17, 99% CI .10-.25) and that reliance on mainstream print predicted a decrease in their belief (β=–.08, 99% CI –.14 to –.02). Although many social media platforms reported downgrading or removing false or misleading content, ongoing use of such platforms by respondents predicted growth in conspiracy beliefs as well (β=.072, 99% CI .018-.123). Importantly, conspiracy belief changes related to media use between the two waves of the study were associated with the uptake of mask wearing and changes in vaccination intentions in July. Unlike other media, use of mainstream broadcast television predicted greater mask wearing (β=.17, 99% CI .09-.26) and vaccination intention (β=.08, 95% CI .02-.14), independent of conspiracy beliefs.

**Conclusions:**

The findings point to the need for greater efforts on the part of commentators, reporters, and guests on conservative media to report verifiable information about the pandemic. The results also suggest that social media platforms need to be more aggressive in downgrading, blocking, and counteracting claims about COVID-19 vaccines, claims about mask wearing, and conspiracy beliefs that have been judged problematic by public health authorities.

## Introduction

At the outset of the COVID-19 pandemic in the United States, various conspiracy theories about the origins and prevention of COVID-19 began to circulate on social media and some conservative media outlets [1-4]. The study of conspiracies as explanations for major events achieved notice in Hofstadter’s classic and influential 1966 volume *The Paranoid Style in American Politics* [5]. In his analysis, conspiracy beliefs presupposed a “vast, insidious, preternaturally effective international conspiratorial network designed to perpetrate acts of most fiendish character” [5]. Conspiracy beliefs, including those claiming the US government’s responsibility for the assassination of John F Kennedy and the 9/11 terrorist attack [6,7], have been a focus of study in the political science literature, which regards such beliefs as ones “in which the ultimate cause of an event is believed to be due to a plot by multiple actors working together with a clear goal in mind, often unlawfully and in secret” [8]. In psychology, the focus has been on understanding what draws individuals to conspiracies [7,9], while in public health, the concern has been about their role in minimizing the likelihood of preventive behavior [10], and their creation of unfounded fears of interventions such as fluoridation and vaccination [11]. As with all conspiracy theories, it is difficult to determine the validity of those related to COVID-19 because the putative actors work in secret [12]. Although stigmatized as paranoid by some [5], such beliefs have a surprising ability to attract adherents [6], and their influence has increasingly been observed in response to COVID-19 public health recommendations, such as vaccination and social distancing [13,14].

A notable characteristic of conspiracy beliefs is the tendency for belief in any one to be associated with acceptance of others [2,15-17]. In the United States, three such beliefs prevalent early in the pandemic [1,2,4] concerned suspicions that the pandemic was the result of malign actions by either the Chinese government or the pharmaceutical industry or that some in the US government were exaggerating the danger of COVID-19 to undermine the president of the United States. A national probability sample of the US population in March and again in July of 2020 [18] found that belief in any one of the conspiracies was highly related to belief in the others and that those beliefs were stable over time. Furthermore, belief in a composite of the three conspiracies in March predicted unwillingness in July to obtain a vaccine for the virus should one become available. The beliefs also predicted a lower likelihood of reporting wearing a face mask outside the home when exposed to other people [18]. Although belief in pandemic conspiracies increased from March to July 2020, our earlier analysis did not identify potential sources of that increase or their possible effects on preventive behavior.

Previous research has found that both misinformation [19] and conspiracy beliefs are resistant to change [6,7,20,21] and that holding conspiracy beliefs related to COVID-19 is associated with lower levels of behaviors known to prevent its spread [13,22]. In addition, single cross-sectional studies have found a positive relationship between social media use and COVID-19 misinformation [1] and conspiracy beliefs [14,23,24] and found that mainstream media consumption is associated with greater rejection of them [25,26]. However, such cross-sectional data cannot determine whether persistent use of these sources is related to change in these beliefs across time or whether efforts undertaken between the two surveys by media outlets to decrease the amount of conspiracy content about COVID-19–related topics is associated with a decrease in these beliefs.

Using a longitudinal study design, we tested the possibility that exposure to different types of media sources might be responsible for change in COVID-19 conspiracy beliefs in the United States. Where our earlier work found that conspiracy beliefs were positively related to use of social and conservative media and negatively related to use of mainstream television and print [18], we sought to determine whether those media sources were also associated with subsequent change in the strength of conspiracy beliefs from March to July 2020.

In examining the role of the media, it is important to recognize that different types of outlets follow different norms when communicating information even in ordinary times. According to normative models of news reporting [27,28], journalists draw on reliable, predictable sources, such as government agencies and other accounts that can be independently verified through standard fact-checking procedures. In the case of conspiracy theories, such evidence is not available [29]. At the same time, their imperviousness to disconfirmation reduces the likelihood that mainstream news sources will feature them except to debunk them. However, commentators on conservative cable and talk radio, and those who post on social media are not bound by such conventions [27]. As one example, Tucker Carlson of Fox News noted that there was “a lot of speculation” that COVID-19 “is not a naturally occurring virus; that it was somehow created by the Chinese government” [30]. Rush Limbaugh alleged that “the coronavirus is being weaponized as yet another element to bring down Donald Trump” and that “it probably is a ChiCom laboratory experiment that is in the process of being weaponized” [31].

Misleading information percolated through social media as the pandemic unfolded [14,23]. On March 3, 2020, NewsGuard [32] raised an alarm due to the following finding:

Over the last 90 days, posts from the websites of the U.S. Centers for Disease Control and Prevention and the World Health Organization received 364,483 ‘engagements,’ or likes, shares, and comments on social media. In that same period, 74 U.S. sites that NewsGuard found to have published coronavirus misinformation received a combined 52,053,542 ‘engagements’—more than 142 times the engagement of the two major public health institutions providing information about the outbreak.

A video on a YouTube channel named the Next News Network was viewed nearly 7 million times before it was taken down by YouTube [33]. It claimed that the COVID-19 pandemic was the result of a deceptive plot that sought to impose “mandatory vaccines” on the public.

Unlike conservative cable and talk radio, the major social media platforms took active measures to interdict COVID-19–related misinformation and conspiracies by removing misleading and potentially harmful content, inserting warnings where appropriate, and featuring articles that refuted the widespread misinformation. In March 2020, Twitter announced that it would “prioritize removing content when it has a clear call to action that could directly pose a risk to people’s health or well-being” [34] and in May announced that it would “put labels and warning messages on some tweets that contain disputed or misleading information related to Covid-19” [35]. In addition, in anticipation of the release of a COVID-19 conspiracy film, “Pinterest had its moderators run proactive searches for terms that might have been associated with the movie, deleting them to nip any problematic content in the bud” [36]. In early March, Facebook announced that it “was removing false claims and conspiracy theories flagged by global health organizations and the company is blocking people from running ads that try to exploit the fears of the public by pitching snake oil cures” [37]. In mid-April, Facebook began “showing messages in News Feed to people who have liked, reacted or commented on harmful misinformation about COVID-19 that we have since removed. These messages will connect people to COVID-19 myths debunked by the WHO including ones we’ve removed from our platform for leading to imminent physical harm” [38]. At the same time, it announced that it had “added a new section to [its] COVID-19 Information Center called Get the Facts. It includes fact-checked articles from [Facebook’s] partners that debunk misinformation about the coronavirus.” In August 2020, Facebook reported that it had removed “7 million posts pushing covid-19 misinformation from its main social media site and Instagram between April and June” and “put warning notes on 98 million covid-19 misinformation posts on Facebook” in the same period [39]. In December 2020, YouTube reported that it had removed more than 700,000 misleading COVID-19 videos to date [40].

Despite their efforts, the range of social media and the capacity of misinformation purveyors to repost interdicted content means that conspiracy theories about COVID-19 often gain substantial audiences before they are blocked or addressed [19,38,41,42]. For example, before YouTube removed a video asserting that the pandemic had been bioengineered, 570,000 subscribers to the website SGT Report had potentially been exposed to it [43]. Additionally, the 26-minute viral video, “Plandemic,” which was also eventually removed in May 2020, nevertheless claimed that “vaccines kill millions, the flu vaccine contains the coronavirus, and that the virus was ‘manipulated’” [44], and was viewed “more than eight million times on YouTube, Facebook, Twitter and Instagram, and had generated countless other posts,” in a little over a week after its release [45]. If exposure to those media is partly responsible for the dissemination and credibility of conspiracy beliefs, we would expect that ongoing exposure would intensify the beliefs between March and July 2020. If the social media efforts to blunt the effects of the content were successful, we would expect that the beliefs reported in March by users of those media would not have intensified by July.

We also were interested to see whether mainstream media use predicted declines in conspiracy beliefs, as cross-sectional analyses of the relations between media sources and conspiracy beliefs in March found [1,18]. If the mainstream news media are a source of substantiated information rather than speculation about conspiracies, then one would expect that exposure to them would result in a decline in conspiracy beliefs regarding the pandemic. Such a finding would suggest that news media can play a role in reducing the strength of conspiracy beliefs, despite their resistance to refutation.

To further investigate the role of conspiracy beliefs on preventive behavior, we asked whether increased belief in COVID-19–related conspiracies from March to July 2020 was related to changes in either preventive behavior (mask wearing) or willingness to accept a COVID-19 vaccine when a safe and effective one becomes available. Furthermore, to the extent to which ongoing use of any media sources was associated with strengthened belief in the same pandemic conspiracies, we were also interested to see if patterns of media exposure might not only predict change in conspiracy beliefs, but also whether those changes were associated with changes in vaccination intentions or with uptake of mask wearing.

In summary, we explored whether use of different media that span the liberal to conservative political spectrum, including mainstream media, predicted change in the acceptance of conspiracy theories that were found to be prevalent at the start of the COVID-19 pandemic in the United States [1,2,4]. In particular, we tested our major hypothesis (H1) that use of mainstream news media such as broadcast television and newspapers that abide by journalistic norms of reporting would predict reduction in conspiracy belief. With regard to social media, which are not constrained by such norms and have been found to be associated with belief in conspiracy theories about the pandemic [14,23], we explored whether their use might have strengthened conspiracy beliefs or alternatively, consistent with efforts by major platforms to remove misinformation and conspiracies, have reduced the strength of those beliefs over time. Our prior analysis of the role of conservative media suggested our second major hypothesis (H2) that use of conservative media would strengthen conspiracy beliefs. Finally, to the extent that media use predicted continued growth or decline in conspiracy beliefs, we asked whether such changes were associated with changes in accepting a vaccine when one became available or with adopting the newly recommended preventive action of mask wearing.

## Methods

### Survey Sample

A sample of US residents (N=840) that was recruited from a national probability panel by Qualtrics completed two waves of an online survey as part of their participation in NORC (National Opinion Research Center) at the University of Chicago’s AmeriSpeak Panel [46]. The first survey was conducted in March 2020 and the second approximately four months later in July. We restricted our analysis to the respondents who completed both surveys (see [18]). The sample that remained at the second wave was very similar to that from the first wave and missingness was only slightly related to intention to vaccinate at wave 1. The study was deemed to be exempt from institutional review board review in as much as no personally identifiable information was retained from the survey firm.

NORC also provided demographic survey weights to enable projection to the US population according to age, gender, race/ethnicity, education, and Census Division based on the Current Population Survey of February 2020. Those weights were applied for descriptive purposes in describing belief in conspiracy theories, but all multivariate analyses were conducted with unweighted data and demographic differences controlled.

A power analysis conducted prior to the study indicated that a sample size of approximately 800 would enable us to detect standardized mediated relationships of .04 or greater at the 99% CI [18], which was regarded as sufficiently sensitive for direct relationships as well.

### Survey Content

#### Conspiracy Beliefs

We assessed belief in three conspiracy theories circulating in social media and other venues at the time of the first wave [47], which were found to be accepted by at least 10% of the US population [1,2,4]:

1. “The pharmaceutical industry created the coronavirus to increase sales of its drugs and vaccines.”
2. “The coronavirus was created by the Chinese government as a biological weapon.”
3. “Some in the U.S. Centers for Disease Control and Prevention, also known as CDC, are exaggerating the danger posed by the coronavirus to damage the Trump presidency.”

Belief in each conspiracy theory was registered on a 4-point scale ranging from “Definitely false” to Definitely true.” Belief in conspiracies is distinguishable from endorsement of other forms of misinformation (eg, that taking vitamin C protects one from contracting COVID-19) that were also prevalent during the early phase of the pandemic in the United States [48]. We operationalized strengthening of conspiracy belief as a movement toward either “definitely” or “probably true” and weakening as movement toward “definitely false.” These ratings were correlated within respondents with values ranging from .40 to .54 at both waves and high levels of reliability (α values=.71 and .74 and ω values=.67 and .74). The mean of these ratings increased from 1.75 (SD 0.85) to 1.90 (SD 1.08), *P*<.001, at the second wave. Belief in the conspiracies in March was inversely related to taking preventive action and accepting an eventual vaccine [18]. In addition, conspiracy belief in March prospectively predicted both action taken and change in vaccination intention assessed in July. In the analysis reported here, we determined whether change in conspiracy belief associated with media use was also related to prospective prediction of those outcomes.

#### Mask Wearing and Other Preventive Actions

We also developed an index encapsulating adoption of seven recommended actions to prevent the infection at wave 1 (eg, frequent hand washing) [18]. The index was the sum of the actions taken in the past few days (Yes or No). This index did not include mask wearing, which was not recommended until after the first wave was completed. At the second wave, we used this outcome as a measure of compliance with recommended action since it had become recognized as critical to halting the spread of the virus through the air as well as preventing contact between the hands and face [49]. At the second wave, 79% (n=668) of the sample reported wearing a mask every day they went to public places where they “might encounter other people.” The two outcomes were positively correlated across the two waves (*r*=.23), indicating that the index at wave 1 was sensitive to willingness to comply with recommended behavior, and so we used the index at wave 1 to assess change in this tendency over time.

#### Vaccination Intentions

We also assessed intentions to accept a vaccine should one become available in the future with a 4-point scale going from “not at all likely” to “very likely.” The proportion who reported either not at all likely or not very likely increased from 14.5% (n=121) at the first wave to 25.8% (n=215) at the second wave, *P*<.001.

#### Media Use

To determine whether media use predicted change across time in conspiracy beliefs, we assessed reliance on six sources of news and commentary in the media from across the political spectrum [11]. Our use of conservative, mainstream, and liberal media categories was consistent with classifications that are commonly used to categorize media in the United States [27,50-52].

For each source, respondents rated “How much information do you get from sources such as…” on a 6-point scale from “no information” to “a lot of information” (see Table 1 for each list of sources). The modal response was “no information” except for mainstream broadcast television news, for which the mode was the highest level of information. We refer to the use of major national newspapers and news services that have users both online and in traditional print as mainstream print. As seen in Table 1, the reported amount of information that respondents received from each source tended to decline from March to July and did so reliably for all but liberal news and aggregators. Nevertheless, individual use of each media category was highly stable across time, as evidenced by the correlations between the two time points. In addition, correlations between use of each media and belief in COVID-19 conspiracies ranged from a high of .37 for conservative media to –.28 for mainstream print.

**Table 1:** Use of six media (with examples of each) as sources of information in both March (T1) and July (T2) of 2020 and relations with political ideology (unweighted).

Media use	March 2020 (T1), mean (SD)	July 2020 (T2), mean (SD)	*t* value of paired difference, T1-T2	Probability of *t* value	Correlation over time	Correlation with T1 Conspiracy Belief Index
Mainstream television (eg, ABC News, CBS News, NBC News)	2.94 (1.77)	2.61 (1.73)	6.13	<.001	.62	–.18
Conservative media (eg, Fox News, Rush Limbaugh, Breitbart News, One America News, The Drudge Report)	1.49 (1.83)	1.39 (1.74)	2.19	.029	.71	.37
Liberal media (eg, MSNBC, Bill Maher, Huffington Post	1.35 (1.53)	1.31 (1.51)	0.69	.49	.57	–.10
Aggregators (eg, Google News, Yahoo News)	2.00 (1.63)	1.94 (1.59)	1.22	.22	.54	.06
Social media (eg, Facebook, Twitter, YouTube)	1.80 (1.71)	1.67 (1.63)	2.23	.026	.54	.16
Mainstream print (eg, Associated Press, New York Times, Washington Post, Wall Street Journal)	1.85 (1.75)	1.80 (1.71)	2.26	.026	.61	–.28

We controlled for the partisanship of the respondent as an alternative explanation for effects, using a measure of political ideology registered on a 5-point scale ranging from “very conservative“ to “very liberal.” As expected, self-identified liberals reported greater use of liberal media (*r*=.30), while conservatives reported greater use of conservative media (*r*=–.42).

We also assessed various demographic differences that could be associated with media exposure and therefore might explain changes between the two waves in relation to conspiracy beliefs and media use. The relations between these characteristics and belief in coronavirus conspiracies are described in the Results section. The text of the survey is located in Multimedia Appendix 1.

### Analysis

We used the program Mplus [53] to test path models for the relation between media use in March 2020 and changes in conspiracy beliefs in July 2020. For any media use that predicted such change, we examined whether the change in conspiracy belief mediated changes in either preventive action (mask wearing) or intention to vaccinate. Less than 3% of the data was missing for any analysis, and Mplus imputed those scores using maximum likelihood estimation. We used bootstrap procedures with 1000 samples to construct 99% and 95% CIs for all tests of direct and mediated paths. We report standardized coefficients for all paths in the models that had CIs excluding zero. To avoid overfitting, paths with values that did not lie outside of at least 90% CIs were dropped from the models and fixed at a value of zero. We used standard measures of goodness of fit for all models [54].

## Results

### Overview

Table 2 shows the percentages of respondents at each wave who reported belief in the three conspiracy theories by various demographic characteristics and media use (weighted to national demographics). Overall, each belief received greater endorsement at the second wave, and this occurred across most of the demographic and media use characteristics. In total, belief that China had created the virus as a weapon was the most widely endorsed (38.1%), with belief that some within the Centers for Disease Control and Prevention (CDC) were using the pandemic to undermine the president was close behind (32.2%). The belief that the pharmaceutical industry was benefiting from the pandemic and possibly helped to create it was the least accepted (17.4%).

**Table 2:** Percentages of sample believing that each conspiracy belief was either “definitely true” or “probably true” by demographic characteristics and news use in March and July 2020 (N=840, weighted).

Characteristic		Percentage of sample	Pharmaceutical industry created the virus, %			Centers for Disease Control and Prevention wants to damage the Trump presidency, %			Chinese government created the virus, %	
			March	July	March		July	March		July
**Gender**										
	Male	44.1	9.6	14.2	27.0^a^		38.2^a^	29.1		37.6^a^
	Female	55.6	19.1	19.8	20.3^a^		27.0^a^	27.8		38.2^a^
**Age**										
	18-29	20.7	26.9^a^	24.4^a^	22.7		26.9	35.1^a^		43.0^a^
	30-44	23.8	21.9^a^	30.7^a^	30.1		34.9	29.8^a^		36.1^a^
	45-59	24.5	10.9^a^	12.9^a^	25.1		36.9	30.3^a^		33.4^a^
	≥60	31.0	4.3^a^	5.9^a^	17.1		30.1	22.3^a^		32.0^a^
**Race/ethnicity**										
	White	74.7	9.7^a^	14.2^a^	22.3^a^		35.5^a^	25.5^a^		38.0
	Black	13.9	36.3^a^	29.1^a^	31.4^a^		18.4^a^	43.8^a^		48.5^a^
	Hispanic	15.4	29.1^a^	28.4^a^	28.6^a^		28.5^a^	33.8^a^		42.9^a^
**Education**										
	High school or less	32.9	27.0^a^	29.0^a^	31.8^a^		42.3^a^	42.7^a^		58.1^a^
	Some college	48.2	12.1^a^	14.0^a^	21.7^a^		30.7^a^	24.0^a^		32.8^a^
	Postgraduate	19.0	6.9^a^	6.4^a^	12.1^a^		17.9^a^	12.7^a^		16.7^a^
**Income (US $)**										
	<30,000	26.4	27.4^a^	22.9^a^	27.2		24.0^a^	37.0^a^		46.8^a^
	30,000-85,000	41.1	13.7^a^	22.0^a^	21.5		36.9^a^	28.5^a^		39.9^a^
	>85,000	32.4	5.6^a^	7.1^a^	22.5		33.1^a^	21.4^a^		28.5^a^
**Political ideology**										
	Conservative	29.8	11.1^a^	17.1^a^	40.2^a^		63.1^a^	37.1^a^		54.8^a^
	Neither	40.0	16.4^a^	17.2^a^	22.6^a^		23.6^a^	30.6^a^		39.9^a^
	Liberal	30.2	16.2^a^	17.5^a^	8.0^a^		13.5^a^	17.4^a^		19.5^a^
**News source**										
	Mainstream television	41.9	13.3	18.3^a^	15.1^a^		17.7^a^	24.7^a^		33.2^a^
	Conservative	18.1	13.4	28.2^a^	33.5^a^		61.1^a^	51.6^a^		65.8^a^
	Liberal	11.1	19.6	22.7^a^	11.0^a^		17.4^a^	21.8		34.1
	Aggregators	20.9	24.2^a^	25.4^a^	25.0^a^		23.6^a^	32.2		43.0^a^
	Social	19.6	19.8^a^	27.1^a^	28.6		32.7	41.8^a^		44.7
	Mainstream print	21.0	15.0^a^	11.5^a^	13.2^a^		11.6^a^	18.4^a^		21.4^a^
Total		100	14.8	17.4	23.5		32.2	28.3		38.1

^a^Response distributions that are significantly different within each time period (*P*<.05) either across demographic groups or within each media use.

**Figure 1:**
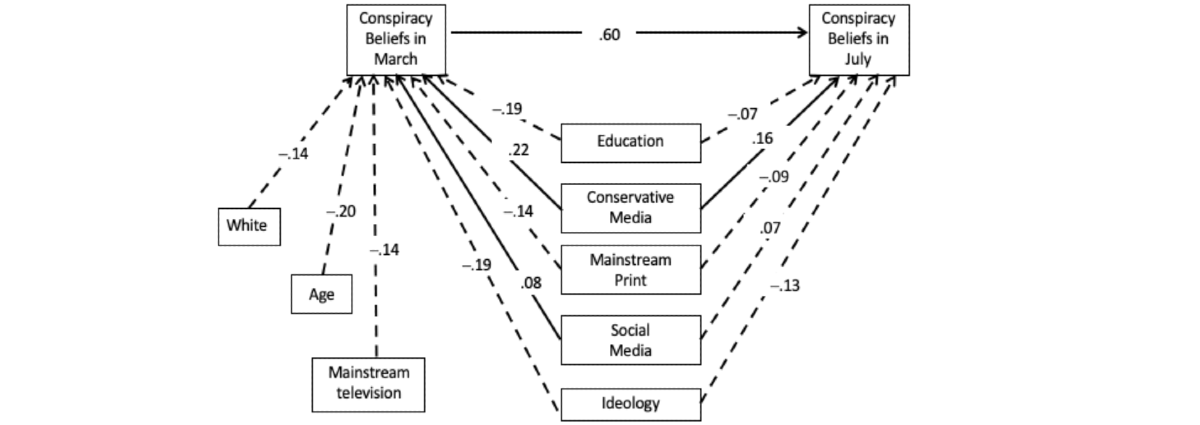
Standardized predictors of conspiracy beliefs in March and again in July with all coefficients falling within 99% CIs, except for social media and conspiracy beliefs in March.

Figure 1 shows the results of the path model that tested our hypotheses regarding mainstream and conservative media as predictors of change in support of pandemic conspiracies and our exploration of social media as a continued influence. The model provided an excellent fit to the data, with a root mean square error of approximation (RMSEA) of .032, 90% CI .000-.068; comparative fit index (CFI)=.99; Tucker-Lewis Index (TLI)=.99, and standardized root mean square residual (SRMR)=.007. As seen in Figure 1, holding constant the relation between conspiracy beliefs across time, our first hypothesis (H1) regarding the effects of mainstream news was only partially supported, while H2 regarding the effects of conservative and social media was fully supported: both conservative (.159, 99% CI .089-.231) and social media use (.072, 99% CI .018-.123) predicted increased belief in the conspiracies. However, while mainstream television and print were inversely related to conspiracy belief in March, only mainstream print predicted a decline in conspiracy belief in July (–.090, 99% CI –.150 to –.029). No other media use predicted subsequent conspiracy belief.

As seen in Figure 1, the media sources remained predictive despite controls for other factors, such as education and liberal political ideology, which predicted declines in conspiracy beliefs.

### Relations With Mask Wearing in July

**Figure 2:**
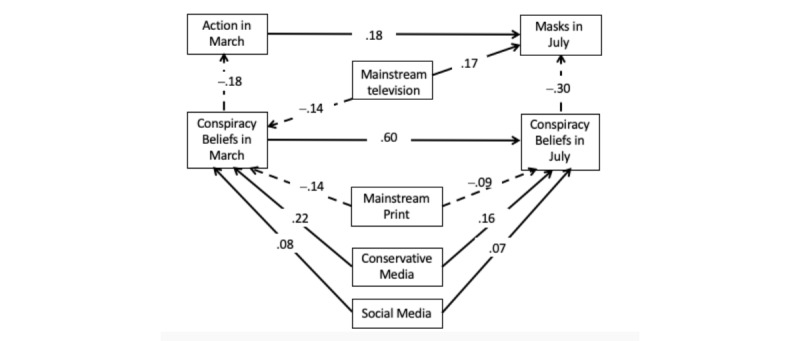
Standardized relations between media use in March and subsequent conspiracy beliefs and action taken to prevent spread of COVID infection in July. All paths within 99% CIs, except between social media and conspiracy beliefs in March, which excluded zero with a 95% CI.

Figure 2 shows the results of the analysis for changes in action taken, with mask wearing specifically assessed at the second wave. This model also fit the data well, with an RMSEA of .036, 90% CI .021-.051; CFI=.98; TLI=.97; and SRMR=.019. Use of masks in July was inversely related to conspiracy belief in July (–.30, 99% CI –.38 to –.22), controlling for demographic differences and political ideology (not shown in the figure). Interestingly, use of mainstream television in March was a positive predictor of mask wearing in July (.17, 99% CI .09-.26), but this relation was direct and not mediated by conspiracy belief in July. This was in contrast with conservative media use in March, which indirectly predicted lower mask wearing in July with an overall relation of –.093, 99% CI –.132 to –.061. Importantly, about half of this mediated relation (–.047/–.093=51%) was attributable to change in conspiracy beliefs (–.047, 99% CI –.076 to –.024). The rest of the relation went through the carryover of conspiracy belief from March to July (–.039, 99% CI –.061 to –.021) and the carryover of preventive action in March to mask wearing in July (–.007, 99% CI –.015 to –.003). Thus, these indirect paths show that use of conservative media in March predicted less mask wearing as mediated by increases in conspiracy belief in July apart from any change attributable to previous actions taken.

With regard to use of mainstream print in March, the overall relation with mask wearing in July was positive (.057, 99% CI .030-.086), indicating that it was associated with greater use of this behavior. In addition, in contrast with conservative media use, its relationship with mask wearing as mediated by change in belief in pandemic conspiracy theories was positive (.027, 99% CI .008-.042). This amounted to about 47% (.027/.057) of the relation between use of mainstream print in March and mask wearing in July. The rest of the relation was mediated by carryover in conspiracy belief from March to July (.026, 99% CI .008-.043) and through action taken in March (.005, 99% CI .001-.010).

Social media use in March also predicted less mask wearing in July with a total indirect relation of –.039, 99% CI –.067 to –.018. Here more than half of this relation (–.022/–.039=56%) was attributable to change in conspiracy beliefs (–.022, 99% CI –.040 to –.006).

### Relations With Vaccination in July

**Figure 3:**
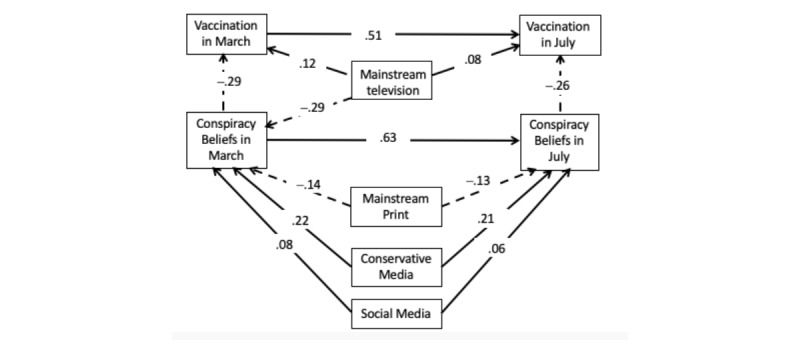
Standardized relations between media use in March and conspiracy beliefs and vaccine intentions in July. All paths within 99% CIs, except between social media and conspiracy beliefs in March and July, which excluded zero at the 95% CI.

Figure 3 shows the model that explored the role of various media uses in March as predictors of change in vaccination intentions and associated change in conspiracy beliefs. This model also fit the data well, with an RMSEA of .058, 90% CI .046-.072; CFI=.97; TLI=.94, and SRMR=.021.

As with mask wearing, vaccination intentions in July were inversely related to conspiracy beliefs in July apart from demographic variables and political ideology (–.26, 99% CI –.34 to –.17). Again, use of mainstream television was a positive predictor in July even though its relation was independent of belief in pandemic conspiracies (.075, 95% CI .016-.135). In the case of vaccination, mainstream television was also associated with greater intention to vaccinate in March (.124, 99% CI .031-.211).

Use of conservative media in March predicted reduced intentions to vaccinate in July as mediated in total by conspiracy beliefs and carryover in vaccination intentions (–.120, 99% CI –.165 to –.083). Use of conservative media in March was specifically related to change in vaccination intentions stemming from changes in conspiracy beliefs in July (–.054, 99% CI –.083 to –.031). This indicates that about 45% (–.054/–.120) of the overall relation was attributable to conservative media’s association with increased belief in pandemic conspiracies from March to July. The rest of the relation was attributable to carryover in conspiracy beliefs from March to July (–.035, 99% CI –.059 to –.019) and carryover from vaccination intention in March to July (–.031, 99% CI –.050 to –.018).

As with mask wearing, use of mainstream print had an overall positive relation with vaccination intention in July (.078, 99% CI .037-116). About 44% (.034/.078) of this relation was due to mainstream print’s association with change in conspiracy belief (.034, 99% CI .017-.055). The rest of the mediation was attributable to carryover in conspiracy beliefs from March to July (.023, 99% CI .007-.040) and carryover in vaccination intentions from March to July (.021, 99% CI .007-.036).

Use of social media in March was also predictive of vaccination in July, with an overall negative indirect relation of –.041, 99% CI –.071 to –.014. However, this relation as mediated by change in conspiracy beliefs was weaker than for the other media, with only about 39% (–.016/–.041) attributable to this source (–.016, 99% CI –.035 to –.001). The rest of the relation was attributable to carryover in conspiracy beliefs and vaccination intentions from March to July.

## Discussion

### Principal Findings

Despite the characterization of conspiracy beliefs as paranoid [5] and stigmatized by some in public discourse [6], they remain robust sources of skepticism regarding important public health recommendations able to prevent the spread of COVID-19. In our analysis of the prevalence of three conspiracy beliefs regarding the COVID-19 pandemic, we found that acceptance of those beliefs ranged from 17% for belief that pharmaceutical companies created the virus to 38% for belief that the Chinese government did so. These levels of acceptance grew over the period from March to July. As with many conspiracies, these beliefs can be accepted by the same person despite their logical incompatibility [55,56]. Scholars have argued that it is the underlying distrust of governments rather than the consistency of their content that appears to motivate their acceptance [57,58].

We found that reliance on different types of media for information during the early months of the COVID-19 pandemic in the United States predicted changes in the strength of belief in the three prevalent conspiracy theories. In particular, use of conservative media, such as Fox News and the talk radio program hosted by the late Rush Limbaugh, was associated with increased acceptance of the three conspiracy beliefs, while use of mainstream print—such as the New York Times and the Wall Street Journal—was associated with increased rejection of them. Although mainstream broadcast television news was negatively associated with conspiracy beliefs in March, there was no direct relation in July. Despite the efforts of the platforms to interdict such content, there was also a small but reliable increase in the perceived truth of the three theories among users of social media in July.

We found partial support for the hypothesis (H1) that use of mainstream news outlets would reduce belief in conspiracy theories because those media tend to follow traditional journalistic norms of reporting based on substantiated information backed by credible sources [27,28]. Conspiracy theories are by their nature spread by questionable sources whose assertions are difficult to verify [29]. As a result, those theories are less likely to be covered in the news, except to undercut them (eg, NBC news coverage of the QAnon conspiracy [59]). This hypothesis was supported in regard to mainstream print, but not broadcast television. We also found support for the hypothesis (H2) that use of conservative media would sustain and strengthen conspiracy beliefs because the commentators on these venues discussed those theories as plausible causes of the pandemic and explanations for failures of the Trump administration to cope with the crisis [60]. As one would expect, we found that, regardless of media use patterns, politically conservative more so than liberal or independent respondents endorsed the conspiracy beliefs at both times. Early in the pandemic, a Republican member of Congress wore a gas mask to a vote on emergency relief for the pandemic, in an apparent attempt to poke fun at the need for such action [61]. Moreover, President Trump opined that the virus was no more serious than the seasonal flu [62], suggested that it was created at the Wuhan Institute of Virology [63], characterized a finding by the Department of Veteran Affairs that hydroxychloroquine was not effective as a “Trump enemy statement” [64], and alleged that the “deep state” was delaying progress on a vaccine to thwart his reelection [65]. These moves to downplay the seriousness of the pandemic, blame its origins on a Chinese lab, and dismiss inconvenient science as the work of his enemies are consistent with two of the conspiracy beliefs on which we focused. Indeed, both the belief that some in the CDC were exaggerating the seriousness of the pandemic in order to undermine his presidency and that the Chinese created the virus as a weapon were endorsed by 63% and 55% of conservative respondents at the second wave, respectively. Conservatives were previously found to be more disposed to accept the COVID-19 conspiracy belief that “powerful people intentionally planned the COVID-19 outbreak” [66].

We did not make a directional prediction about the influence of social media on continued belief in conspiracy theories because, as we noted earlier, during the period between our two surveys, considerable effort was expended by major platforms to remove or downgrade misleading information about the pandemic [40,41]. In addition, a recent survey of content on Twitter concluded that despite the large amount of misinformation on social media, there is also a great amount of science-based information that circulates on those sites [67]. However, our findings suggest that these efforts did not remove the influence of the three theories on which we focused. This finding is consistent with evidence from a study conducted in April 2020 that found considerable evidence of tweeted comments supporting a conspiracy theory about the pandemic [68] and other work suggesting that conspiracy claims that are interdicted quickly reappear elsewhere [17,45]. Although experience in China suggests that rapid refutation of pandemic rumors can be successful in reducing transmission of misinformation on social media [69], the expanse, variety, and decentralized nature of US media make such efforts more challenging than in countries whose media are largely government controlled. We also found that acceptance of conspiracy theories was associated with less action to prevent the spread of the disease, such as mask wearing. Similar patterns were observed in willingness to accept a vaccine. In both cases, use of conservative and social media were related to reductions in preventive behavior associated with changes in conspiracy beliefs.

Although it is difficult to debunk conspiracy theories, we did find that use of mainstream print predicted a subsequent decrease in the three beliefs. In addition, persons with greater education were more likely to move away from belief in those theories by July. Nevertheless, despite the potential ability of mainstream print to reduce belief in pandemic conspiracies, its influence appeared to be weaker than that of conservative and social media, which together predicted more than twice the effect size of mainstream print (.23 versus –.09). If this pattern of influence were to persist, it could lead to further increases in conspiracy belief among users of social and conservative media and, because of the smaller influence of print, a net increase in those beliefs in the US population at large.

On a positive note, the influence of mainstream television news, which also reaches a more sizeable audience than social and conservative media, appeared to outweigh their potentially negative effects on mask wearing and future vaccination. Indeed, mainstream television was the most used source of information in our sample. Despite the greater *change* in conspiracy beliefs associated with conservative and social media use, when the direct relations with vaccination intentions (.08) and mask wearing (.17) are included, the difference between social and conservative versus mainstream media use was negligible for vaccination (–.161 versus .158, respectively) and weaker for mask wearing (–.132 versus .227, respectively). Considering the larger reach of mainstream news, the overall potential effects of those news sources could well have outweighed the effects of conservative and social media use on the public’s acceptance of vaccination and mask wearing.

Some have suggested that use of mainstream news is so dominant in the United States that disinformation transmitted through the internet and social media is unlikely to exert much influence [70]. Our findings are consistent with the conclusion that although mainstream news use is extensive and correlated with positive protective behaviors, the influence of social and conservative media is nonetheless significant. The three conspiracy beliefs that we studied are associated with media use outside of mainstream news, suggesting that these sources have a worrisome influence on the US public, despite their smaller share of the media market.

It is notable that use of mainstream television news was associated with greater mask wearing and intentions to vaccinate whether one accepted the three conspiracies or not. Use of broadcast television was also associated with taking recommended action in the United Kingdom [26] and Canada [25]. This pattern suggests that this popular source of information enhances compliance with recommended behavior by means other than dispelling conspiracy theories. Through its wide reach and its audiovisual capacities, it may provide direct exposure to persons wearing masks and taking vaccines, actions that would increase the normative acceptance of these behaviors. It is also likely that mainstream news sources such as television transmit more public health recommendations than are routinely accessed on social media [25] or conservative cable and opinion sites [60]. Trust in mainstream news is also associated with greater rejection of conspiracy theories [71].

It is noteworthy as well that some individual characteristics were no longer directly related to increased belief in COVID-19 conspiracies in July despite being associated with them in March. Although both Black and Hispanic respondents reported more belief in those theories than white respondents in March, Black and Hispanic respondents did not increase their belief by the second wave in July. This suggests that they were not exposed to greater sources of COVID-19 conspiracy information in the media during the intervening period. Importantly, neither Black nor Hispanic respondents were more likely to consume information on conservative media (*r* values ranging from –.015 to .057) and were only somewhat more likely to use information on social media (*r* values ranging from .017 to .099). At the same time, Black respondents were more likely to use mainstream television than others at both time points (*r* values=.167 and .144), while Hispanic respondents exhibited no differential use of information on any of the two remaining media groupings over the two time points. Nevertheless, despite the absence of an increase in conspiracy belief among Black and Hispanic respondents, their greater overall endorsement of such beliefs is still related to their unwillingness to accept vaccines.

### Limitations

Although our two-wave panel study provides more sensitive evidence of the role of media use in the persistence of conspiracy beliefs regarding the COVID-19 pandemic, there are limitations that need to be recognized. We use a national probability sample to generalize to the US population, but the survey was conducted online, which restricted the sample to those with online access and experience. As a result, older citizens were underrepresented. Additionally, because we rely on self-reports of behavior, such as mask wearing, we cannot confirm that those reports reflect actual behavior. Our results also are only applicable to changes that occurred between March and July of 2020 in the United States. The effects of media use on pandemic conspiracy beliefs beyond that period remain to be studied. Finally, despite the ability to observe changes in conspiracy beliefs associated with media use, we cannot make strong causal claims because it still remains possible that characteristics other than those for which we controlled drove the changes in those beliefs. Nevertheless, our controls for a wide range of demographic differences as well as for political ideology increase our confidence that media use predicts changes in conspiracy beliefs regarding the pandemic.

### Conclusions

Exposure to conservative and social media during the period from March to July 2020 predicted greater belief in three COVID-19 conspiracy beliefs in the United States, and these beliefs were related to less intention to vaccinate in the future and lower reported use of masks in the present. Public health agencies tasked with communicating the need for effective action to prevent the spread of the virus should seek opportunities to present accurate information about the pandemic to users of those media. At the same time, reaching users of mainstream media is also important in that they were either less likely to subscribe to conspiracy beliefs (in the case of print) or more likely to adopt protective behavior (in the case of mainstream broadcast television news). Although social media platforms have attempted to remove misinformation and conspiracy theories related to the COVID-19 pandemic, users of these platforms were more likely to exhibit increases in the strength of such beliefs in July. This finding suggests that those venues need to exert even greater efforts to counteract exposure to problematic COVID-19–related content.

## Appendix

Multimedia Appendix 1 Questionnaire items for national survey.

## Abbreviations

CDC: Centers for Disease Control and Prevention
CFI: comparative fit index
RMSEA: root mean square error of approximation
SRMR: standardized root mean square residual
TLI: Tucker-Lewis Index
