# Supplementary material for: Patterns of Media Use, Strength of Belief in COVID-19 Conspiracy Theories, and the Prevention of COVID-19 From March to July 2020 in the United States: Survey Study
Source: J Med Internet Res. 2021 Apr 27;23(4):e25215. doi: 10.2196/25215 (PMC8083953; doi:10.2196/25215)
Supplement: Multimedia Appendix 1 [file jmir_v23i4e25215_app1.docx]

**Romer & Jamieson Survey Administered in March and July of 2020**

Introduction: We are conducting a research study on behalf of the University of Pennsylvania to learn how people are responding to the coronavirus (COVID-19) outbreak.

The survey will take about 10 minutes to complete.  The information you give will be kept confidential and will not be linked to your name. All data we collect will be de-identified and stored for future research.

If there is a question you wish not to answer, then you may skip it.  You may withdraw your consent and discontinue participation at any time

How likely, if at all, do you think you or someone in your family will become infected with the coronavirus (COVID-19)?

- Very likely (4)
- Somewhat likely (3)
- Not too likely (2)
- Not at all likely (1)
- I have been infected with the coronavirus (7)
- Someone in my family has been infected with the coronavirus (8)

How worried, if at all, do you feel about the possibility that you or someone in your family will become infected with the coronavirus (COVID-19)?

- Very worried (4)
- Somewhat worried (3)
- Not too worried (2)
- Not at all worried (1)
- I have been infected with the coronavirus (7)
- Someone in my family has already been infected by it (8)

In the past few days, which of the following, if any, have you done to protect yourself from getting the coronavirus (COVID-19)? (MARK ALL THAT APPLY)

NOTE: At the second wave, this was asked on a three-point scale going from Never, Some days, to Every day.

- Washed hands or used a hand sanitizer (1)
- Avoided gatherings greater than 10 people (2)
- Avoided touching nose, eyes, and mouth with unwashed hands (3)
- Kept distance between yourself and other people (4)
- Worn a face mask (5)
- Covered nose and mouth with tissue or used the inside of your elbow when sneezing or coughing (6)
- Avoided close contact with people who are sick (7)
- Stayed at home (8)
- Taken the test for coronavirus (9)
- Other (Specify) (97) ________________________________________________
- Nothing (0)

If there were a vaccine that protected you from getting the coronavirus (COVID-19) how likely, if at all, would you be to decide to be vaccinated?

- Very likely (4)
- Somewhat likely (3)
- Not too likely (2)
- Not at all likely (1)

For each of the following statements, please indicate whether you believe it is true or false.

|  |
| --- |

The pharmaceutical industry created the coronavirus (COVID-19) to increase sales of its drugs and vaccines. Do you believe this is

- Definitely true (4)
- Probably true (3)
- Probably false (2)
- Definitely false (1)

|  |
| --- |

Some in the U.S. Centers for Disease Control and Prevention, also known as the CDC, are exaggerating the danger posed by the coronavirus (COVID-19) in order to damage the Trump presidency. Do you believe this is

- Definitely true (4)
- Probably true (3)
- Probably false (2)
- Definitely false (1)

The coronavirus (COVID-19) was created by the Chinese government as a biological weapon. Do you believe this is

- Definitely true (4)
- Probably true (3)
- Probably false (2)
- Definitely false (1)

How much information do you get from sources such as Fox News, Rush Limbaugh, Breitbart News, One America News or The Drudge Report?

|  | NO information (0) | (1) | (2) | (3) | (4) | A LOT of information (5) |
| --- | --- | --- | --- | --- | --- | --- |
| Sources such as Fox News, Rush Limbaugh, Breitbart News, One America News or The Drudge Report (1) |  |  |  |  |  |  |

How much information do you get from sources such as MSNBC, Bill Maher or the Huffington Post?

|  | NO information (0) | (1) | (2) | (3) | (4) | A LOT of information (5) |
| --- | --- | --- | --- | --- | --- | --- |
| Sources such as MSNBC, Bill Maher or the Huffington Post (1) |  |  |  |  |  |  |

How much information do you get from sources such as ABC News, CBS News or NBC News?

|  | NO information (0) | (1) | (2) | (3) | (4) | A LOT of information (5) |
| --- | --- | --- | --- | --- | --- | --- |
| Sources such as ABC News, CBS News or NBC News (1) |  |  |  |  |  |  |

How much information do you get from sources such as Google News or Yahoo News?

|  | NO information (0) | (1) | (2) | (3) | (4) | A LOT of information (5) |
| --- | --- | --- | --- | --- | --- | --- |
| Sources such as Google News or Yahoo News (1) |  |  |  |  |  |  |

How much information do you get from sources such as Facebook, Twitter or YouTube?

|  | NO information (0) | (1) | (2) | (3) | (4) | A LOT of information (5) |
| --- | --- | --- | --- | --- | --- | --- |
| Sources such as Facebook, Twitter or YouTube (1) |  |  |  |  |  |  |

How much information do you get from sources such as the Associated Press, The New York Times, the Washington Post, or the Wall Street Journal?

|  | NO information (0) | (1) | (2) | (3) | (4) | A LOT of information (5) |
| --- | --- | --- | --- | --- | --- | --- |
| Sources such as the Associated Press, The New York Times, or the Washington Post (1) |  |  |  |  |  |  |

Generally speaking, would you describe your political views as:

- Very liberal (5)
- Somewhat liberal (4)
- Moderate (3)
- Somewhat conservative (2)
- Very conservative (1)

The remaining questions will help us understand the people who took part in our survey.

What is your gender identity?

- Male (1)
- Female (2)
- Other (7) ________________________________________________

What is your age?

________________________________________________________________

Could you please tell me if you are between the ages of:

- 18-24 (1)
- 25-29 (2)
- 30-49 (3)
- 50-60 (4)
- 61-64 (5)
- 65 or older (6)

Are you of Hispanic or Latino background, such as Mexican, Puerto Rican, Cuban or other Spanish background?

- Yes (1)
- No (2)

How would you describe your race?

- White (1)
- Black or African-American (2)
- Asian (3)
- American Indian (4)
- Some other race (7) ________________________________________________
- Mixed race (6)

What is the highest level of school you have completed or the highest degree you have received?

- Less than high school (Grades 108 or no formal schooling) (1)
- High school incomplete (Grades 9-11 or Grade 12 with NO diploma) (2)
- High school graduate (Grade 12 with diploma or GED certificate) (3)
- Some college, no degree (includes some community college) (4)
- Two-year Associate's degree from a college or university (5)
- Four-year college or university degree/Bachelor's degree (e.g.: BS, BA, AB) (6)
- Some postgraduate or professional schooling, no postgraduate degree (7)
- Master's degree (e.g.: MA) (8)
- Doctorate degree (e.g.: PhD) (9)
- Professional degree, such as a medical or law degree (e.g.: MD, JD) (10)

Those are all the questions I have for you. 


That completes our survey.  Thank you for participating.  If you have any additional questions about the study, you may contact the project investigator at the University of Pennsylvania at APPCdatasets@appc.upenn.edu


Thank you very much for your time and cooperation.  Have a nice day/evening.
